# Acetaminophen and Tetracycline Removal Using *Prosopis juliflora*-Derived ZnO-Modified Biochar: Evaluation in Batch and Continuous Systems

**DOI:** 10.1021/acsomega.5c12318

**Published:** 2026-03-16

**Authors:** Manjunath Singanodi Vallabha, Syeda Rabia Asma, Rajkumar Reddy, Bhojaraja Mohan, Chikmagalur Raju Girish

**Affiliations:** † Department of Civil Engineering, 93092 B.M.S. College of Engineering, Bangalore 560019, Karnataka, India; ‡ Department of Chemical Engineering, Manipal Institute of Technology, 76793 Manipal Academy of Higher Education, Manipal 576104, Karnataka, India

## Abstract

The discharge of pharmaceutical active compounds (PhACs) into aquatic environments has become a growing concern due to their adverse effects on both aquatic organisms and human health. Simultaneously, the global spread of invasive weeds disrupts ecosystems, leading to significant environmental and economic consequences. This study investigates competitive adsorption of acetaminophen (ACT) and tetracycline (TET) using green-synthesized ZnO-biochar derived from *Prosopis juliflora* (ZPJC). ZPJC was characterized using SEM, EDX, FTIR, XRD, TGA, and pH_pzc_ analyses and applied in both batch and column experiments for monocomponent (ACT/TET) and multicomponent (ACT+TET) systems. Batch experiments examined the impact of operational parameters such as initial PhAC concentration (0.1–10 mg/L), contact time (1–180 min), pH (3–11), and ZPJC dose (0.25–4 g/L). Column experiments explored the variations in bed depth (3–9 cm), flow rate (0.5–2 L/h), and influent concentration (1–5 mg/L). Optimal conditions (60 min, 6.5 pH, and 3 g/L ZPJC dose) resulted in a maximum adsorption capacity of 5.27 mg/g for TET and 9.26 mg/g for ACT in the batch system, following pseudo-second-order and Langmuir models, suggesting chemisorption dominance. For column systems, the Thomas and Yoon–Nelson models better represented experimental data. Adsorption efficiency improved with increasing bed depth and flow rate, while it declined with higher PhAC concentration. In multicomponent batch systems, TET exhibited antagonistic effects due to site competition and steric effects, whereas ACT demonstrated slight synergism. However, in column systems, both ACT and TET displayed antagonistic interactions. Scale-up design of the column elucidates that ZPJC can be adopted as a sustainable solution for the removal of PhACs while addressing invasive weed proliferation.

## Graphical abstract

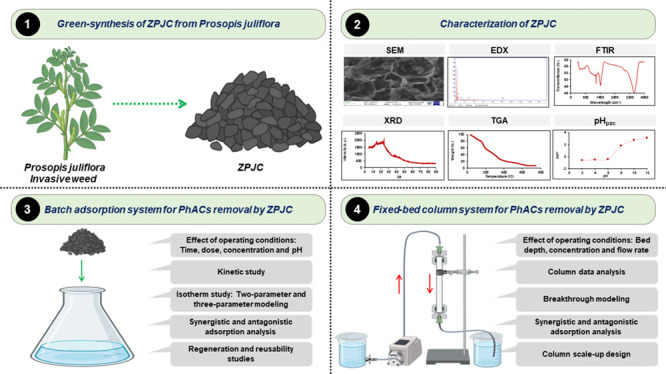

## 1 Introduction

Pharmaceutical active compounds (PhACs) found in medicines, personal care products, and disinfectants are increasingly detected in water bodies due to improper disposal, human excretion, and effluent discharges.^1^ Their presence poses environmental and health risks as conventional treatment systems often fail to remove these compounds effectively.^2^ Prolonged exposure may cause antibiotic resistance, endocrine disruption, organ toxicity, allergic reactions, reproductive and developmental disorders, and even cancer risks.^3^

Tetracycline (TET) and acetaminophen (ACT) are extensively consumed PhACs worldwide, frequently detected in aquatic environments, and have distinctly different physicochemical characteristics. TET is a broad-spectrum antibiotic widely used in human and veterinary medicine, aquaculture, and livestock production, and its persistent release into the environment has been associated with the emergence of antimicrobial resistance and disruption of aquatic ecosystems.^4^ On the other hand, ACT, a commonly used analgesic and antipyretic, is one of the most heavily consumed pharmaceuticals worldwide and is routinely detected in surface waterbodies and effluents due to incomplete removal in conventional treatment systems.^5^ Both compounds exhibit significant persistence and mobility in aquatic environments, facilitating their accumulation in water bodies and potential transfer through food chains. Importantly, their contrasting molecular weights, functional groups, polarity, and solubility characteristics (Table S1) make them suitable model pollutants for systematically evaluating the adsorption performance of the developed adsorbent under both single- and multipollutant conditions.

Advanced wastewater treatment is essential to mitigate PhACs effectively.^6^ Among the available methods such as oxidation, biodegradation, adsorption, and membrane filtration, adsorption is considered most promising due to its efficiency at low concentrations, operational simplicity, low energy requirements, and potential for adsorbent regeneration.^7^ Unlike other processes, adsorption does not generate excess sludge or demand excessive chemicals, making it more sustainable and eco-friendly.^8^ The eco-friendly nature of adsorption is strengthened when biochar from invasive weeds is used as an adsorbent, enabling waste valorization and reducing reliance on synthetic materials. Sustainability is further enhanced through green synthesis approaches, where biochar and biochar-based composites are prepared by using low-energy processes and environmentally benign reagents. These methods minimize secondary pollution while improving the adsorption performance.

Over the past few decades, biochar has gained attention as an adsorbent for its abundance, low cost, and ability to be produced from agricultural and industrial residues. Additionally, using invasive weed biomass to produce biochar not only supports wastewater treatment but also aligns with multiple sustainable development goals (SDGs). Specifically, it supports SDG 6 by eliminating pollutants and ensuring access to safe water while also contributing to SDG 7 by reducing energy consumption in treatment processes compared with other technologies. Moreover, it enhances sustainable water management and aligns with broader sustainability objectives. By promoting responsible resource utilization (SDG 12) and environmentally friendly production methods (SDG 13), biochar plays a crucial role in sustainability. It further safeguards aquatic ecosystems from PhACs (SDG 14) and promotes sustainable land management by controlling proliferation of invasive weeds (SDG 15).^9,10^

Numerous studies have explored different adsorbents for removing PhACs from single-pollutant systems, including materials such as orange peel,^1^ ZnAl-coated bagasse biochar,^4^ *Azadirachta indica*-coated ZnO nanoparticles,^11^ Fe_3_O_4_^−^ coated coffee residue,^12^ bamboo charcoal,^13^ and *Prosopis juliflora*-activated carbon.^14^ In the meantime, *Prosopis juliflora*, an invasive species native to Mexico found across different parts of the world, including India, is considered a noxious weed due to its negative economic and environmental impacts. It disrupts native ecosystems, depletes water resources, and deteriorates soil quality, posing a significant threat to agriculture.^15^ Converting *Prosopis juliflora* biomass into biochar presents a sustainable approach to managing invasive weeds while also creating added value.

Despite substantial research on PhACs removal, limited studies have explored the simultaneous removal of multiple PhACs, particularly using biochar modified with green-synthesized nanoparticles. Green synthesis employs biological sources such as plant extracts or microorganisms to produce metal oxide nanoparticles in an eco-friendly and cost-effective manner, minimizing toxic chemical use and enhancing sustainability.^16^ *Prosopis juliflora* leaf extract contains bioactive compounds such as flavonoids, phenolics, alkaloids, saponins, and tannins that act as reducing, stabilizing, capping, and nucleating agents for ZnO nanoparticle formation.^17,18^ Previous studies mainly targeted PhACs in monopollutant systems using batch processes, leaving a gap in research on multipollutant adsorption. Competitive (antagonistic) or cooperative (synergistic) interactions of multiple PhACs during adsorption are rarely addressed. Furthermore, fixed-bed column studies, more representative of real-scale applications, remain limited, with most investigations restricted to batch setups.

The main aim of this study is to investigate the potential of green-synthesized ZnO-coated biochar derived from *Prosopis juliflora* (ZPJC) for adsorption of TET and ACT from both single (ACT/TET) and multipollutant (TET + ACT) systems. The scope of study includes the following: (i) synthesis of ZPJC and characterization using various techniques, (ii) conduct batch adsorption experiments to investigate the influence of time, dose, concentration, and pH on the adsorption of ACT and TET, (iii) analyze the adsorption kinetics and equilibrium using various kinetic and isotherm models, (iv) assess the dynamic adsorption performance of ZPJC in a fixed-bed column by examining the effects of flow rate, bed depth, and concentration, and (v) apply breakthrough curve models for interpreting column behavior and performance under dynamic conditions and develop scale-up design for column system, emphasizing on critical parameters such as adsorption efficiency and bed utilization capacity.

## 2 Materials and Methods

### 2.1 Chemicals

TET (C_22_H_24_N_2_O_8_·HCl; MW: 480.90 g/mol) and ACT (C_8_H_9_NO_2_; MW: 151.163 g/mol) of analytical grade were purchased from SRL chemicals and HiMedia, India, respectively. Other analytical grade chemicals like zinc nitrate hexahydrate [Zn­(NO_3_)_2_·6H_2_O], sulfuric acid (H_2_SO_4_), hydrochloric acid (HCl), sodium hydroxide (NaOH), potassium hydroxide (KOH), isopropyl alcohol (C_3_H_8_O), methanol (CH_3_OH), and acetonitrile (CH_3_CN) were obtained from SD. Fine Chem Ltd., Isochem, and Sigma-Aldrich, India. All chemicals were used as received without any further purification or treatment.

### 2.2 Synthesis of Adsorbent

Synthesis of ZPJC involved three stages: (i) preparation of *Prosopis juliflora* biochar (PJC), (ii) green synthesis of ZnO nanoparticles using *Prosopis juliflora* leaf extract, and (iii) immobilization of ZnO onto PJC to produce ZPJC.

#### 2.2.1 Preparation of PJC

*Prosopis juliflora* biomass was collected near agricultural land, Bangalore, India, cut into 2–3 cm, washed, and shade-dried for 10 days. The dried material was treated with 1:1 (w/w) HCl for 6 h, rinsed with deionized water, and oven-dried at 80 °C overnight. It was then pyrolyzed at 300 °C for 2 h in a muffle furnace, cooled, crushed to 1–2 mm, and labeled as PJC.

#### 2.2.2 Green Synthesis of ZnO

20 g of dried *Prosopis juliflora* leaves were boiled in 1 L of distilled water for 10 min and filtered. Then, 100 mL of leaf extract was mixed with 4 g of zinc nitrate hexahydrate and stirred at 1500 rpm at 60 °C until a thick yellow paste was formed. This paste was then calcined at 200 °C for 2 h to yield ZnO nanoparticles.^19^

#### 2.2.3 Synthesis of ZPJC

500 mg of green-synthesized ZnO was dispersed in 250 mL of distilled water and stirred for 20 min. Then, 5 g of PJC was added and stirred at 250 rpm for 4 h at room temperature. The mixture was filtered, washed, and dried at 150 °C overnight.^20^ The resulting ZPJC was stored in an airtight container for adsorption studies.

### 2.3 ZPJC Characterization

Surface morphology of ZPJC was examined using high-resolution SEM (Carl Zeiss EVO 10, Germany), and elemental composition analysis was conducted using energy-dispersive X-ray diffraction (EDX, Tescan Vega3LMU, Czech Republic). Crystalline or amorphous nature was determined using X-ray diffractometry (PanAnalytical Xpert Pro). Thermal stability of ZPJC was assessed using thermogravimetric analysis (TGA, Mettler Toledo, USA), while Fourier transformation infrared (FTIR, PerkinElmer, USA) radiation spectrophotometry was used to identify the functional groups present on the ZPJC surface. Point of zero charge (pH_pzc_) was determined following established literature methods.^21^

### 2.4 Experimental Methodology

Experiments were conducted using ZPJC in both batch and fixed-bed column systems to evaluate the removal of pollutants from monopollutant (ACT/TET) and multipollutant (ACT + TET) systems.

#### 2.4.1 Adsorption in Batch System

Batch adsorption experiments were conducted in 250 mL conical flasks containing either ACT or TET solution placed on an orbital stirrer operating at 150 rpm at room temperature (25 ± 2 °C) to evaluate the effect of various operational parameters. The study investigated the effect of ACT/TET concentrations ranging from 0.1 to 10 mg/L, contact time between 0 and 180 min, solution pH from 3 to 11, and ZPJC dosages from 0.25 to 4 g/L. All experiments were performed in duplicates (*n* = 2).

Following adsorption, ACT and TET concentrations were measured using high-performance liquid chromatography (HPLC, Shimadzu, Japan). Stock solutions of ACT and TET (100 mg/L) were prepared and diluted to obtain the required working concentrations. Quantification was performed using an HPLC system equipped with a reverse-phase C18 column. ACT was analyzed using a methanol:water (60:40 v/v) mobile phase at 1 mL/min, 20 μL injection volume, and detection at 254 nm (retention time ∼ 4.2 min). Meanwhile, TET analysis employed an acetonitrile:0.1% formic acid (50:50 v/v) mobile phase, with detection at 360 nm (retention time ∼ 6.5 min).

Adsorption capacity (*q*_e_, mg/g) and removal (%) were calculated using eqs 1 and 2, respectively.

$$q_{e}=[C_{0}-C_{e}]\times \frac{V}{m}$$ 1

$$\mathrm{Removal}\;(\%)=\left[\frac{C_{0}-C_{e}}{C_{0}}\right]\times 100$$ 2

where *C*_0_ and *C*_e_ are the initial and equilibrium concentrations (mg/L), *V* is the solution volume (L), and *m* indicates the ZPJC mass (g).

#### 2.4.2 Adsorption in a Fixed-Bed Column System

Fixed-bed column experiments for TET and ACT removal were conducted using an acrylic column (1 cm internal diameter, 25 cm height). ZPJC was packed between 1 cm layers of glass wool and glass beads to ensure stability and uniform flow. ACT/TET solution was introduced from the top using a peristaltic pump (Ravel, India), and treated samples were collected at the outlet for analysis.

Bed depth (*H*) was examined at 3, 6, and 9 cm, with 2.5 mg/L concentration and 1 L/h flow rate. Flow rate influence was assessed by varying it at 0.5, 1, and 2 L/h with a fixed-bed depth of 6 cm and a concentration of 2.5 mg/L. Subsequently, the effect of ACT/TET concentration was studied at 1, 2.5, and 5 mg/L while keeping 6 cm depth and 1 L/h flow rate.

### 2.5 Modeling of Adsorption Data

#### 2.5.1 Kinetic Modeling

Kinetic study was conducted in 250 mL flasks containing 200 mL of ACT/TET solution (10 mg/L and 6.5 ± 0.3 pH). Optimal contact time and ZPJC dose were applied. Samples collected at different intervals were analyzed using kinetic models: pseudo-first-order, pseudo-second-order, liquid-film diffusion, intraparticle diffusion, and Elovich models (Table S2).

#### 2.5.2 Equilibrium Modeling

Equilibrium studies were performed in 250 mL flasks with 100 mL of ACT/TET solutions (0.1–10 mg/L at 6.5 ± 0.3 pH). Optimal contact time and ZPJC dose were applied. Adsorption mechanism and maximum adsorption capacity were evaluated using two-parameter (Langmuir, Freundlich, Temkin, Dubinin–Radushkevich, and Elovich) and three-parameter (Redlich–Peterson, Khan, Hill, Toth, and Sips) isotherm models (Table S2).

All kinetic and isotherm parameters were determined using nonlinear regression analysis of experimental adsorption data. The quality and reliability of model fitting were evaluated using multiple statistical indicators, including coefficient of determination (*R* ^2^) and error functions such as the chi-square test (Χ^2^) and root-mean-square error (RMSE).

#### 2.5.3 Breakthrough Curves Modeling

Breakthrough models (Thomas, Yoon–Nelson, Adams–Bohart, and BDST) were applied to analyze the breakthrough curves under varying conditions (Table S3). Adsorption parameters calculated including cumulative adsorbate mass retained (*m*_ads_, mg), equilibrium sorption capacity (*q*_e_, mg/g), total influent adsorbate mass (*m*_total_, mg), effluent volume (*V*_eff_, mL), empty bed contact time (EBCT, min), removal (*R*, %), and mass transfer zone (MTZ, cm) were determined using expressions presented in Table S4.

### 2.6 Competitive Adsorption Analysis

Batch and fixed-bed column experiments evaluated the antagonistic (competitive) or synergistic (cooperative) interactions during ACT and TET removal in multicomponent systems. In the batch mode, competitive adsorption was investigated at varying ACT + TET concentrations (0.1 + 0.1, 0.25 + 0.25, 0.5 + 0.5, 1 + 1, 2 + 2, 4 + 4, 5 + 5, and 10 + 10 mg/L) using an optimized ZPJC dose, with mixtures stirred at 150 rpm until equilibrium. To interpret competitive/cooperative mechanisms, the Langmuir competitive model in eq 3 and its linearized forms for TET and ACT (eqs 4 and 5), respectively, were applied.

$$q_{e,ij}=\frac{q_{m}K_{L}C_{e}}{1+\sum K_{L,ij}C_{e,ij}}$$ 3

$$\frac{1}{q_{e,T}}=\frac{1}{Q_{\max,T}}+\frac{1}{Q_{\max,T}\times K_{L,T}}\left[\frac{1+K_{L,A}\times C_{e,A}}{C_{e,T}}\right]$$ 4

$$\frac{1}{q_{e,A}}=\frac{1}{Q_{\max,A}}+\frac{1}{Q_{\max,A}\times K_{L,A}}\left[\frac{1+K_{L,T}\times C_{e,T}}{C_{e,A}}\right]$$ 5

At equilibrium, the concentration is denoted as *C*_e_ (mg/L) and the adsorption capacity as *q*_e_ (mg/g). The maximum adsorption capacity is *q*_m_ (mg/g), with *K*_L_ as the Langmuir constant. For binary systems (ACT + TET), ‘*i*’ refers to the target pollutant, while ‘*j*’ corresponds to the competing pollutant. *C*_e,T_ and *C*_e,A_ represent the equilibrium concentrations of TET and ACT, with *q*_e,T_, *q*_e,A_, *Q*_max,T_, and *Q*_max,A_ denoting their equilibrium and maximum sorption capacities. Fixed-bed studies use ACT + TET (2.5 + 2.5 mg/L), 6 cm depth, and 1 L/h flow rate.

### 2.7 Regeneration and Reusability Study

After adsorption, ZPJC was allowed to settle, and ACT/TET solutions were replaced with an equal quantity of deionized water to initiate desorption. Flasks were agitated for 120 min, and the samples were analyzed for the desorbed amount of ACT and TET. Furthermore, ZPJC regeneration was assessed over two adsorption–desorption cycles, with desorption efficiency (%) calculated using eq 6.

$$\mathrm{Desorption}\;(\%)=\left[\frac{C_{\mathrm{des}}}{C_{\mathrm{ads}}}\right]\times 100$$ 6

where *C*_des_ (mg/L) indicates the TET/ACT concentration desorbed from ZPJC, and *C*_ads_ (mg/L) represents the TET/ACT concentration adsorbed onto ZPJC.

## 3 Results and Discussion

### 3.1 Characterization of ZPJC

ZPJC characterization is illustrated in Figure 1a–f. The SEM image in Figure 1a reveals a rough, porous surface with interconnected pores (∼4 μm) that facilitate the efficient diffusion of PhACs. The coarse texture suggests successful ZnO incorporation, with uniform distribution across the carbon matrix, potentially enhancing adsorption and photocatalytic behavior. EDX spectrum (Figure 1b) confirms the elemental composition: high carbon content (69.74%) indicating the dominance of the carbon matrix, supporting the adsorption capacity. Oxygen (25.4%), indicating functional groups for chemical interactions, and Zn (3%) verify ZnO loading onto biochar, which can further improve ZPJC adsorption and photocatalytic and antimicrobial performance.

**Figure 1:**
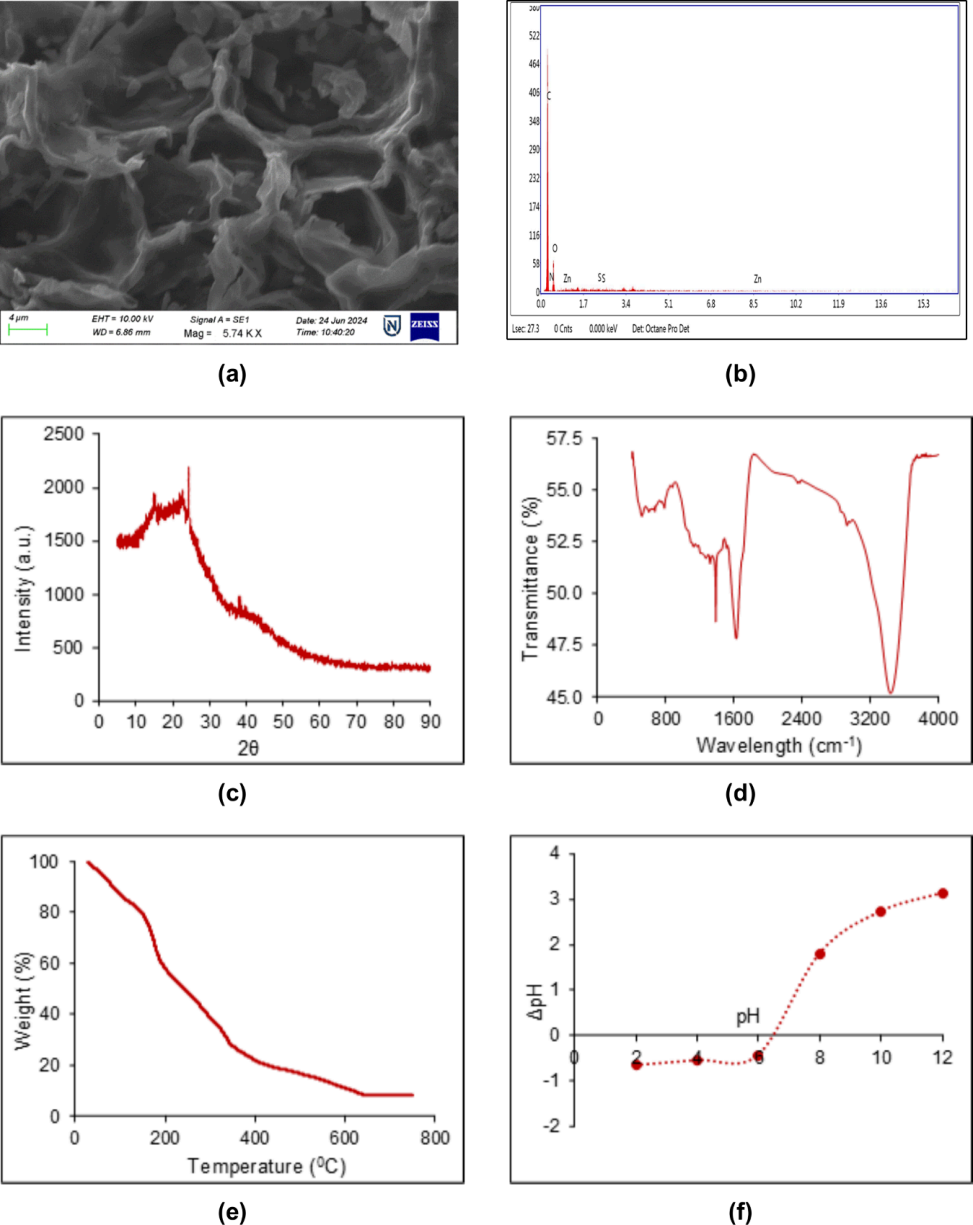
Characterization of ZPJC: (a) SEM, (b) EDX, (c) XRD, (d) FTIR, (e) TGA, and (f) pH_pzc_.

XRD pattern (Figure 1c) exhibits a broad peak at 2θ = 24°, representing amorphous carbon, and a smaller peak at 2θ = 38°, suggesting that ZnO is dispersed in a nanostructured form within the carbon matrix. This dispersion provides more active sites, strengthens ZnO–carbon interactions, and supports pollutant removal through electrostatic interactions, surface complexation, or photocatalytic activity under light. FTIR spectrum (Figure 1d) confirms the functional groups: O–H stretching (3200–3600 cm^–1^), N–H bending (3413 cm^–1^), C=C aromatic stretching (1632 cm^–1^), and C–I stretching (501 cm^–1^). Peaks at 500–700 cm^–1^ confirm ZnO vibrations, validating ZnO incorporation onto the carbon matrix.

TGA analysis (Figure 1e) indicates multiphase decomposition: ∼21.45% mass loss below 150 °C (moisture), ∼68.55% loss between 150 and 600 °C (organic volatilization), and ∼3% loss at 600–800 °C (ZnO and stable residues). Figure 1f shows the pH_pzc_ of ZPJC ∼6.5. Surface is positively charged at pH < 6.5 and negatively charged at pH > 6.5. At neutral pH, ACT (p*K*_a_ 9.5) remains largely neutral, whereas TET (p*K*_a_ 3.3, 7.7, and 9.7) exists in multiple ionic states, favoring sorption.

### 3.2 Batch Adsorption System

Batch experiments assessed key operating parameters, with kinetic and isotherm modeling elucidating ACT and TET adsorption mechanisms.

#### 3.2.1 Effect of Operating Conditions

Figure 2a illustrates the impact of time on adsorption of ACT and TET. Adsorption was rapid during the first 30 min, reaching 84.45% for TET and 86.76% for ACT, attributed to the abundance of available active sites on ZPJC. Adsorption rate then slowed, and equilibrium was achieved within 60 min, with final efficiencies ∼88.29% (TET) and 92.51% (ACT). The slightly higher uptake of ACT is ascribed to the smaller, hydrophilic structure that favors hydrogen bonding with ZPJC, while the steric hindrance from TET’s complex structure limits the interaction.^22^ In Figure 2b, at a lower dosage (0.1–2 g/L), the removal increased notably due to higher availability of active adsorption sites on ZPJC. Beyond 2 g/L, a sharper improvement was observed, reaching ∼87.84% (TET) and 92.08% (ACT) at 3 g/L. This enhancement is linked to greater surface area and more active sites. Further increasing the dosage to 4 g/L resulted in only marginal gains (TET: 88.17% and ACT: 92.37%) as adsorbent sites exceeded available pollutant molecules, leaving many unoccupied.^23^

**Figure 2:**
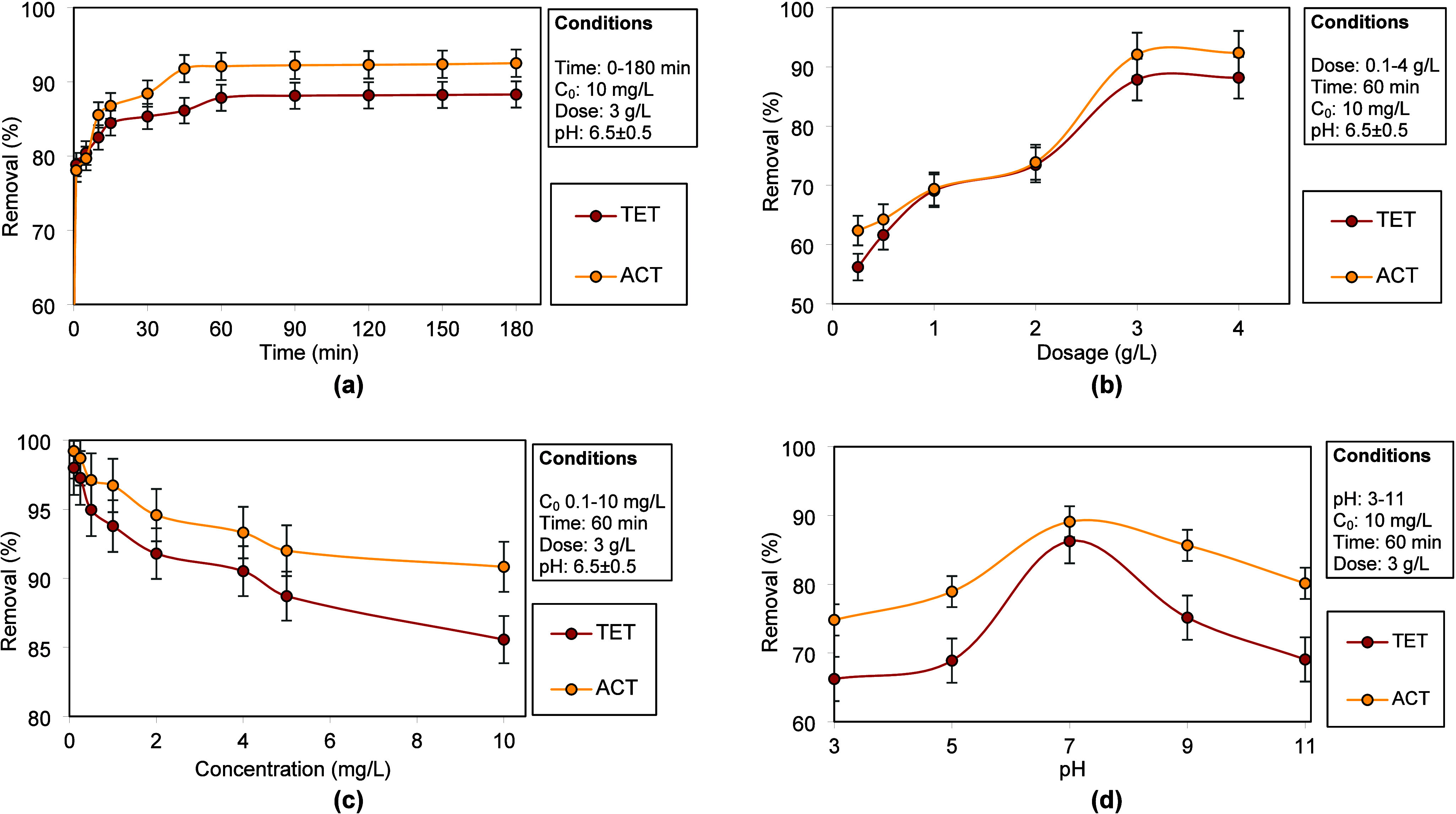
Influence of operating parameters on the adsorption of TET and ACT: (a) time, (b) ZPJC dosage, (c) concentration, and (d) pH.

From Figure 2c, at lower concentrations, abundant sites enabled high adsorption, with nearly complete removal (∼98–99%) of both pollutants. As the concentration increased, competition for limited active sites reduced removal, although the total adsorption increased. At 10 mg/L, the removal drops to 85.6% (TET) and 90.83% (ACT). TET showed greater decline, suggesting that ACT has a stronger affinity for ZPJC.^24^ Figure 2d shows that at acidic pH, removal was reduced, likely due to protonation of the adsorbate and adsorbent functional groups, hindering interactions. Adsorption improved near-neutral pH because the reduced proton competition from H^+^ ions enhanced electrostatic attraction and hydrogen bonding. Beyond neutral pH, the adsorption declined, particularly for TET. ACT (p*K*_a_ 9.5) remains neutral under acidic and near-neutral conditions and shows a maximum uptake at near-neutral pH. At pH > 9.5, deprotonation leads to repulsion with negatively charged ZPJC (pH_pzc_: 6.5), lowering the removal. TET (p*K*_a_ 3.3, 7.7, and 9.7) exhibits charge variability; it is positively charged at pH < 4 (repulsion with ZPJC), zwitterionic near neutrality (maximum removal via hydrogen bonding and hydrophobic effects), and negatively charged above pH 7.7, causing repulsion and reduced adsorption. TET–OH complexation under alkaline conditions further decreases the efficiency. Thus, optimal adsorption for both PhACs occurs around neutral pH, where the charge balance and molecular interactions are most favorable.^24,25^

#### 3.2.2 Kinetic Modeling

Table 1 presents the kinetic parameters for ACT and TET adsorption on ZPJC. Pseudo-first-order model showed a reasonable fit, with modeled adsorption capacities close to experimental values. However, the pseudo-second-order model exhibited higher *R* ^2^ with lower Χ^2^ and RMSE for both pollutants (Table S5), confirming chemisorption as the dominant mechanism. Intraparticle-diffusion model analysis indicated slower diffusion for TET (*K*_ID_: 0.042) compared to ACT (*K*_ID_: 0.082). ACT also showed a boundary layer constant (*C*), suggesting greater surface adsorption, though *R* ^2^ values (0.996 for TET, 0.954 for ACT) suggest that intraparticle diffusion is not the sole rate-controlling step. Liquid-film-diffusion model gave higher diffusion rates (*K*_FD_) for TET, yet results imply that the external mass transfer is secondary. Elovich model fitting revealed a higher initial adsorption rate (α) and desorption constant (β) for ACT, suggesting stronger interactions. Overall, the pseudo-second-order model provided the best fit, with ACT displaying faster and stronger adsorption than TET.

**Table 1:** Kinetic and Isotherm Parameters for TET and ACT Removal Employing ZPJC

type	models	constants	TET	ACT
		*q*_e–exp_	2.93	3.07
kinetic models	pseudo-first-order	*q*_e_	2.800	2.912
		*K*_1_	0.781	0.890
		*R* ^2^	1.000	0.985
	pseudo-second-order	*q*_e_	2.838	2.955
		*K*_2_	3.821	2.101
		*R* ^2^	1.000	0.999
	intraparticle-diffusion	*K*_ID_	0.042	0.082
		*C*	2.603	2.557
		*R* ^2^	0.996	0.954
	liquid-film-diffusion	*A*	2.140	1.980
		*K*_FD_	1.165	0.980
		*R* ^2^	0.992	0.995
	Elovich	α	25.00	30.00
		β	2.500	2.750
		*R* ^2^	0.993	0.924
two-parameter isotherm models	Langmuir	*q*_m_	5.267	9.256
		*K*_1_	0.823	0.579
		*R* ^2^	0.998	1.000
	Freundlich	*K*_F_	2.980	4.192
		1n	0.986	0.934
		*R* ^2^	0.990	0.992
	Temkin	*A*_T_	30.00	275.07
		*B*	0.800	0.349
		*R* ^2^	0.996	0.999
	Dubinin–Radushkevich	*q*_m_	4.993	6.392
		*K*_DR_	0.0002	0.0002
		*R* ^2^	0.981	0.981
	Elovich	*q*_m_	1.609	1.618
		*K*_E_	2.323	3.186
		*R* ^2^	0.970	0.970
three-parameter isotherm models	Redlich–Peterson	*k*	19.86	5.600
		*n*	0.366	0.564
		*a*	7.919	0.687
		*R* ^2^	0.999	0.961
	Khan	*q*_m_	2.990	5.500
		*K*	1.200	0.752
		*n*	0.567	0.274
		*R* ^2^	0.962	0.979
	Sips	*q*_m_	24.96	113.40
		*K*	0.042	0.009
		*n*	0.734	0.745
		*R* ^2^	0.999	0.989
	Hill	*q*_sH_	5.725	7.736
		*n*_H_	0.227	0.294
		*K*_D_	0.335	0.426
		*R* ^2^	0.976	0.969
	Toth	*K*_T_	5.726	7.736
		*A*_T_	0.077	0.161
		*T*_T_	0.052	0.111
		*R* ^2^	0.980	0.971

#### 3.2.3 Equilibrium Modeling

Table 1 presents the isotherm constants for ACT and TET adsorption. The Langmuir model, which assumes monolayer adsorption on homogeneous sites, showed higher maximum adsorption capacity (*q*_m_) for ACT (9.256 mg/g) compared to TET (5.267 mg/g) with strong correlation coefficients. Langmuir separation factor (*R*_L_), defined in eq 7, ranged between 0 and 1 for both pollutants, confirming favorable adsorption.

$$R_{L}=\frac{1}{1+(K_{L}C_{o})}$$ 7

where *K*_L_ indicates the adsorbent–adsorbate’s affinity; *R*_L_ (0–1) signifies favorable, *R*_L_ > 1 suggests unfavorable, and *R*_L_ = 0 indicates irreversible adsorption.

The Freundlich model, describing multilayer adsorption on heterogeneous surfaces, also fitted well (*R* ^2^ > 0.99). Freundlich constant (*K*_F_) was higher for ACT compared to TET, implying greater affinity, while 1n values reflected moderately heterogeneous surfaces for both pollutants. The Temkin model incorporates adsorbate–adsorbent interactions, revealing higher adsorption intensity (*A*_T_) for ACT compared to TET, suggesting stronger binding forces with the ZPJC surface. In contrast, the Dubinin–Radushkevich model indicated low *K*_DR_ values for both ACT and TET, confirming mainly a physical adsorption mechanism, though weak correlations *R* ^2^ limited its applicability. The Elovich model suggested slightly stronger adsorption energy (*K*_E_) for ACT than for TET, indicating stronger interactions between ACT and ZPJC.

Among three-parameter models, the Redlich–Peterson model provided better overall fit by combining the Langmuir and Freundlich characteristics. Adsorption capacity parameter (*k*) was higher for TET than ACT, indicating stronger initial adsorption tendency. The Khan model also indicated higher ACT adsorption capacity, while the Sips model predicted substantially greater adsorption for ACT, further supporting its stronger affinity. The Hill model, which evaluates cooperative binding, showed a higher *n*_H_ value for ACT, reflecting stronger cooperative adsorption. The Toth model, an advanced Langmuir-based model for heterogeneous surfaces, also demonstrated strong agreement with experimental data, with ACT exhibiting higher sorption capacity than TET. Based on higher *R* ^2^ values with lower Χ^2^ and RMSE (Table S5), the Langmuir model was the best-fitting model for experimental data of both pollutants, indicating a monolayer chemisorption mechanism.

### 3.3 Fixed-Bed Column System

Figure 3 shows the breakthrough curves for ACT and TET, while Table S6 presents the corresponding column adsorption data.

**Figure 3:**
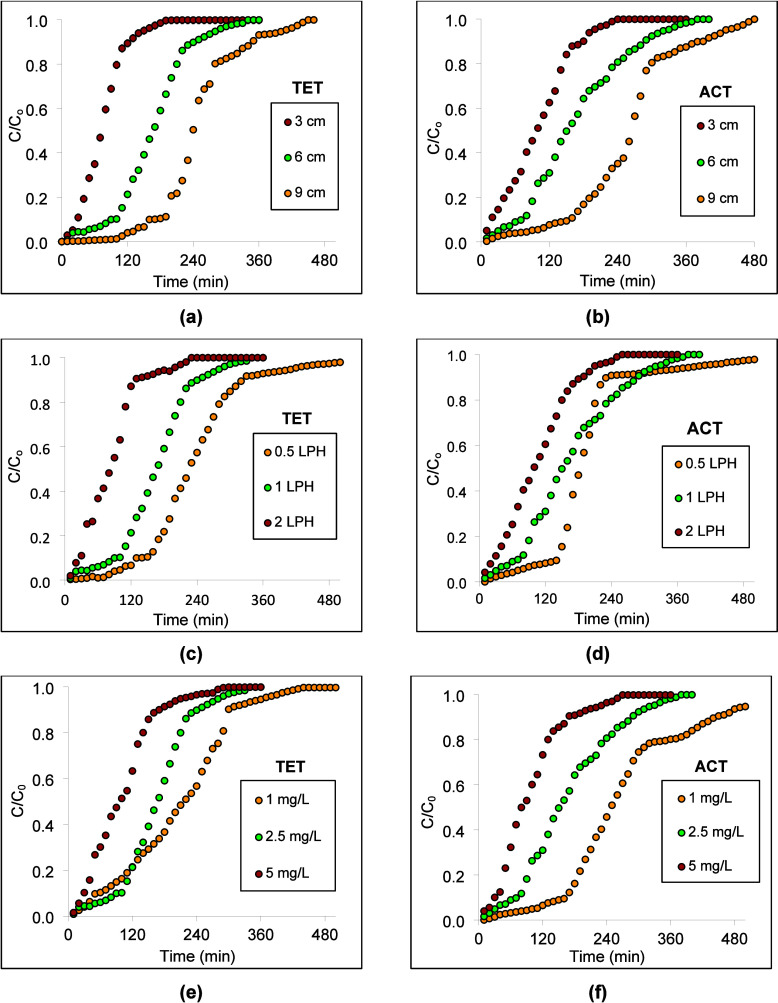
Effect of column operating parameters: bed depth (a) TET and (b) ACT; flow rate (c) TET and (d) ACT; concentration (e) TET and (f) ACT.

#### 3.3.1 Effect of Column Operational Parameters

Figure 3a,b shows the influence of bed depth on ACT and TET adsorption performance. Increasing the bed depth from 3 to 9 cm significantly extended both breakthrough (*C*/*C*_o_ = 0.1) and exhaustion times (*C*/*C*_o_ = 0.9) due to the availability of a large adsorbent surface area and a longer residence time (Table S6). At 1 L/h and 2.5 mg/L, increasing the depth from 3 to 9 cm, the breakthrough time for TET and ACT increased from 30 to 160 min and 20 to 160 min, accompanied by an increase in removal from 57.38 to 70.92% and 54.69 to 65.88%, respectively. Shallow beds exhibited steep breakthrough curves, indicating rapid saturation, whereas deeper beds showed more gradual profiles, reflecting improved mass transfer.^26–28^ Slower breakthrough of ACT compared to TET suggests relatively stronger interactions with ZPJC. Increasing bed depth also resulted in higher EBCT, enhancing the adsorbent-adsorbent contact and more effective utilization of adsorption sites. The associated increase in the MTZ length indicates a broader active adsorption region and more progressive breakthrough behavior.

In Figure 3c,d, reducing the flow rate from 2 to 0.5 L/h at a fixed bed depth of 6 cm and an influent concentration of 2.5 mg/L delayed the breakthrough and improved the removal for both TET and ACT. Lower flow rates increased EBCT by reducing the linear velocity, allowing sufficient time for pollutant diffusion into ZPJC pores. In contrast, higher flow rates shortened the residence time, leading to incomplete mass transfer, earlier breakthrough, and steeper breakthrough curves.^29,30^ MTZ values increased with increasing flow rates, indicating a reduced mass transfer efficiency and faster column exhaustion.

Figure 3e,f shows the effect of influent concentration on the adsorption behavior, indicating a reduced breakthrough time due to rapid occupation of available adsorption sites, with increasing ACT/TET concentration from 1 to 5 mg/L at a fixed-bed depth (10 cm) and flow rate (1 L/h). At lower concentrations, sufficient active sites ensured a higher removal efficiency, whereas higher pollutant loading accelerated saturation and reduced the operational time. Although higher concentrations enhanced the mass transfer driving force and adsorption capacity, they produced sharper breakthrough fronts and larger MTZ, indicating faster exhaustion of the bed.^24,31,32^ Overall, increased bed depth and reduced flow rate favored higher EBCT and improved mass transfer, while higher influent concentration accelerated bed saturation, consistent with fixed-bed principles.

#### 3.3.2 Breakthrough Models Analysis

Table 2 provides the breakthrough model parameters for TET and ACT adsorption on ZPJC. Increasing the bed depth from 3 to 9 cm resulted in a decline in Thomas (*K*_TH_), Yoon–Nelson (*K*_YN_), and Adams–Bohart (*K*_AB_) rate constants, reflecting reduced adsorption rates due to greater mass transfer limitations.^33^ Although deeper beds enhance adsorption zones, they also slow adsorption by introducing additional resistance. Higher flow rates produced opposite effects, with the increase in *K*_TH_, *K*_YN_, and *K*_AB_ indicating faster adsorption kinetics from improved mass transfer. Similarly, increasing the concentration from 1 to 5 mg/L increased *K*_TH_ and *K*_YN_, demonstrating a stronger driving force for adsorption, but decreased *K*_AB_ due to rapid saturation that lowered the overall efficiency.

**Table 2:** Breakthrough Model Parameters for Adsorption of TET and ACT Employing ZPJC

							Thomas			Yoon–Nelson			Adams–Bohart		
system	adsorbate	*H* (cm)	*Q* (L/h)	*C*_0_ (mg/L)	*q*_e,exp_ (mg/g)	τ_0.5–exp_ (min)	*K*_TH_ × 10^–3^ (L/mg min)	*Q*_TH_ (mg/g)	*R* ^2^	*K*_YN_ (1/min)	τ_0.5_ (min)	*R* ^2^	*K*_AB_ × 10^–3^ (L/mg min)	*N*_AB_ (mg/L)	*R* ^2^
monocomponent (TET/ACT)	TET	3	1	2.5	8.7	80	17.28	8.70	0.9820	0.04	77	0.9820	5.64	2847	0.6983
		6	1	2.5	9.64	170	10.48	9.64	0.9903	0.03	165	0.9903	4.52	2430	0.8754
		9	1	2.5	9.66	240	9.88	10.08	0.9840	0.03	259	0.9840	7.84	1810	0.9448
		6	0.5	2.5	6.83	230	7.76	7.38	0.9613	0.02	253	0.9613	4.00	1766	0.7795
		6	2	2.5	9.7	90	12.80	10.01	0.9903	0.03	86	0.9903	4.84	3147	0.8754
		6	1	1	4.79	220	18.30	4.78	0.9373	0.02	205	0.9373	7.80	1242	0.6656
		6	1	5	11.61	100	5.28	12.01	0.9828	0.03	103	0.9828	1.78	4028	0.8250
	ACT	3	1	2.5	11.49	100	11.52	11.28	0.9932	0.03	97	0.9932	4.20	3434	0.8273
		6	1	2.5	9.59	160	8.20	9.87	0.9855	0.02	169	0.9855	3.72	2580	0.7909
		9	1	2.5	9.99	270	7.04	10.01	0.9846	0.02	257	0.9846	4.72	1937	0.9785
		6	0.5	2.5	5.66	190	6.48	5.87	0.8780	0.02	201	0.878	2.80	2385	0.6974
		6	2	2.5	12.08	100	11.04	12.06	0.9855	0.03	103	0.9855	4.28	3558	0.7909
		6	1	1	6.23	250	15.30	6.30	0.9908	0.02	270	0.9908	10.60	1276	0.7995
		6	1	5	11.00	80	5.20	11.38	0.9526	0.03	98	0.9526	1.64	4024	0.8961
multicomponent (TET + ACT)	TET	6	1	2.5 + 2.5	8.41	150	9.64	8.50	0.99	0.02	146	0.99	4.40	2198	0.7909
	ACT				8.69	140	8.32	9.05	0.97	0.02	155	0.97	3.32	2758	0.6479

In general, greater beds reduced rate constants, while they enhanced the adsorption capacity (*Q*_TH_) and breakthrough time (τ_0.5_) because of larger surface area and longer contact. However, the number of adsorption sites (*N*_AB_) declined under these conditions. In contrast, higher flow rates lowered *Q*_TH_ and τ_0.5_, while they increased *N*_AB_, suggesting faster initial uptake with reduced retention. At higher pollutant concentrations, *Q*_TH_ and τ_0.5_ initially rose, but saturation occurred earlier, reducing τ_0.5_ and *N*_AB_.^34^

For ACT and TET, the predicted *Q*_TH_ values closely matched the experimental adsorption capacity (*q*_e,exp_), confirming the reliability of the Thomas model. Likewise, the Yoon–Nelson model-predicted breakthrough times (τ_0.5_) were consistent with experimental times (τ_0.5,exp_). Minor deviations at low depths or high flow rates suggest external film diffusion and pore diffusion effects.^35–38^

Further, BDST model constants (Table S7) indicated that the breakthrough time increased with bed depth, as observed in both experimental and model data. TET exhibits slightly higher *N*_o,_ while ACT showed a marginally higher rate constant. Overall, Thomas and Yoon–Nelson models best described the adsorption behavior, while the Adams–Bohart model was more applicable in describing the initial adsorption phase.

### 3.4 Competitive Adsorption Analysis

The study evaluated adsorption of TET and ACT in the multicomponent system to identify antagonistic or synergistic interactions. Antagonism arises when one pollutant reduces the other’s adsorption through site competition, affinity differences, or solution chemistry effects. In contrast, synergism occurs when one pollutant enhances the adsorption of another by modifying the surface properties or improving the site accessibility.^19^ These effects were quantified using the adsorption capacity ratio $\left[\frac{Q_{\mathrm{Multi}}}{Q_{\mathrm{Mono}}}\right]$ , where $\frac{Q_{\mathrm{Multi}}}{Q_{\mathrm{Mono}}}=1$ indicates no interaction, $\frac{Q_{\mathrm{Multi}}}{Q_{\mathrm{Mono}}}<1$ suggests the antagonistic effect, and $\frac{Q_{\mathrm{Multi}}}{Q_{\mathrm{Mono}}}>1$ implies the synergistic effect.^9^

Results (Table S8) showed that ACT (10.53 mg/g) adsorbed more strongly than TET (2.13 mg/g), confirming ZPJC’s greater affinity for ACT. The $\frac{Q_{\mathrm{Multi}}}{Q_{\mathrm{Mono}}}$ ratio revealed the antagonistic effect for TET (0.40) due to site competition and steric hindrance, while ACT showed a slight synergistic effect (1.14), likely from cooperative interactions. Overall, ZPJC exhibited preferential adsorption of ACT over TET.

Furthermore, column breakthrough profiles (Figure S1) further confirmed these trends: TET exhibits earlier breakthrough and adsorbs less in ACT’s presence, while ACT showed negligible difference between single and binary systems. However, column studies (Table S6) indicated antagonistic adsorption, $\frac{Q_{\mathrm{Multi}}}{Q_{\mathrm{Mono}}}$ for both ACT (0.92) and TET (0.88), likely from reduced mass transfer and competitive effects. Overall, antagonistic effects were stronger for TET, highlighting its lower affinity under a mixed matrix.

### 3.5 Regeneration and Reusability Potential of ZPJC

Figure S2 depicts the desorption behavior over four successive cycles, indicating predominantly reversible interactions for both ACT and TET on the ZPJC surface, with the gradual decline attributed to partial occupation of stronger sites and limited accessibility of molecules retained within inner pore regions. Compared to ACT, TET shows lower desorption efficiency, which may be associated with its relatively larger molecular size and the presence of multiple functional groups that may facilitate stronger and multivalent interactions with surface functional groups and ZnO sites on ZPJC. Such interactions can reduce desorption reversibility. In contrast, ACT, with fewer binding functionalities, may interact more weakly and thus desorb more readily. Despite this difference, sustained desorption over four cycles confirms good regeneration ability of ZPJC for repeated adsorption–desorption applications. Furthermore, desorption may be further improved by using acidic, basic, or organic eluents, which could selectively weaken adsorbate–surface interactions and enhance the regeneration efficiency in repeated cycles.

### 3.6 Mechanism of Adsorption

FTIR spectra (Figure S3) revealed the adsorption of TET and ACT onto ZPJC by showing shifts in functional groups before and after adsorption in both mono- and multicomponent systems. Shift or reduction of the broad band around 3200–3500 cm^–1^, attributed to the stretching of the O–H and N–H bonds, indicated hydrogen bonding and electrostatic interactions. Variations in peaks between 1000 and 1700 cm^–1^ (C=O, C=C, and C–N vibrations) supported additional chemical interactions, while signals in the 500–900 cm^–1^ range suggested possible metal–oxygen coordination. Overall, adsorption involved hydrogen bonding, electrostatic attraction between negatively charged ZPJC and protonated groups of ACT/TET, and π–π stacking from the aromatic rings.

On the other hand, pH strongly influenced the uptake due to ionization states. At low pH, protonation of both pollutants and ZPJC reduced the level of electrostatic interactions. Near-neutral pH, reduced H^+^ competition enhanced the adsorption of both compounds. ACT (p*K*_a_: 9.5) remains neutral under acidic to near-neutral pH, favoring hydrophobic and π–π interactions; above pH 9.5, deprotonation caused electrostatic repulsion with ZPJC (pH_pzc_: 6.5). TET (p*K*_a_: 3.3, 7.7, and 9.7) was repelled at pH < 4, while in the zwitterionic form at neutral pH, it enabled hydrogen bonding and hydrophobic interactions, yielding a maximum uptake. At alkaline pH (pH > 7.7), deprotonation and TET–OH complexation reduced adsorption.

Equilibrium data suggested monolayer adsorption, consistent with chemisorption, further confirmed by pseudo-second-order kinetics. SEM images revealed a porous morphology, indicating pore filling as an additional mechanism. Overall, ACT and TET adsorption occurred via hydrogen bonding, electrostatic interactions, π–π stacking, and pore filling, supported by kinetic, equilibrium, and FTIR analysis (Figure S4).

### 3.7 Design of Scale-Up Column

Scale-up of fixed-bed adsorption column was carried out using characteristic time (t*), defined at 50% breakthrough, corresponding to utilization of approximately half of the adsorbent bed. This approach ensures consistency with laboratory-scale behavior and enables reliable prediction of key performance parameters, including adsorption capacity (AC), utilization capacity (UC), degree of utilization (DOU), length of unused bed (LUB), treated effluent volume (*V*_FO_), and effluent concentration (*C*_avg_).^9^ Table 3 presents the comparison of lab-scale and scaled-up column performance of ZPJC for TET and ACT removal under mono- and multicomponent conditions.

**Table 3:** Scale-Up Design of Column for Removal of TET and ACT Using ZPJC

			monocomponent		multicomponent	
parameters		unit	TET	ACT	TET	ACT
Lab Column (1 cm Φ × 6 cm)						
characteristic time (*t*_lab_^*^)	time when *C*/*C*_o_ = 0.5 (from BT curve)	min	170.00	160.00	150.00	140.00
breakthrough time (*t*_b–lab_)	time when *C*/*C*_o_ = 0.1 (from BT curve)	min	90.00	80.00	70.00	60.00
adsorption capacity of bed (AC)	AC = *Q*_lab_ × *C*_o_ × *t*_lab_^*^	mg	17.34	16.32	15.30	14.28
utilization capacity of bed (UC)	UC = *Q*_lab_ × *C*_o_ × *t*_b–lab_	mg	9.18	8.16	7.14	6.12
degree of utilization (DOU)	DOU = UC/AC × 100	%	52.9	50.0	46.7	42.9
length of unused bed (LUB)	LUB = *L* (1 – *t*_b–lab_/*t*_lab_^*^)	cm	2.82	3.00	3.20	3.43
mass of adsorbate flown out (*M*_FO–lab_)	*M*_FO–lab_ = *M*_total_ – *M*_ads_	mg	3.53	5.24	3.58	5.05
volume flown out (*V*_FO–lab_)	*V*_FO–lab_ = *Q* × *t*_b–lab_	L	1.53	1.36	1.19	1.02
average *C* in effluent (*C*_avg_)	*C*_avg_ = *M*_FO–lab_/*V*_FO–lab_	mg/L	2.31	3.85	3.01	4.95
Desired Scale-Up Column (50 cm Φ × 300 cm)						
characteristic time (*t*_scale_^*^)	*t*_scale_^*^ = *t*_lab_^*^ × (*L*_scale_/*L*_lab_)	day	5.90	5.56	5.21	4.86
breakthrough time (*t*_b–scale_)	*t*_b–scale_ = *t*_scale_^*^ – (*t*_lab_^*^ – *t*_b–lab_)	day	5.85	5.50	5.15	4.81
flow rate (*Q*_scale_)	*Q*_scale_ = *Q*_lab_ × (*A*_scale_/*A*_lab_)	L/min	42.25	42.25	42.25	42.25
adsorption capacity of bed (AC)	AC = *Q*_scale_ × *C*_o_ × *t*_scale_^*^	kg	0.898	0.845	0.792	0.739
utilization capacity of bed (UC)	UC = *Q*_scale_ × *C*_o_ × *t*_b–scale_	kg	0.889	0.837	0.784	0.731
degree of utilization (DOU)	DOU = *t*_b–scale_/*t*_scale_^*^ × 100	%	99.06	99.00	98.93	98.86
length of unused bed (LUB)	LUB = *L*_scale_ × (1 – *t*_b–scale_/*t*_scale_^*^)	cm	2.82	3.00	3.20	3.43
mass of adsorbate flown out (*M*_FO–scale_)	*M*_FO–scale_ = *D*_scale_^2^ × *M*_FO–lab_	kg	0.009	0.013	0.09	0.013
volume flown out (*V*_FO–scale_)	*V*_FO–scale_ = *Q*_scale_ × *t*_b–scale_	m^3^	355	334	313	293
average *C* in effluent (*C*_avg_)	*C*_avg_ = *M*_FO–scale_/*V*_FO–scale_	kg/m^3^	0.025	0.039	0.029	0.043

At the laboratory scale, shorter characteristics and breakthrough times arise from the bed depth and contact time, resulting in moderate AC and UC values and noticeable unused bed. In multicomponent systems, both ACT and TET exhibit earlier breakthrough, lower utilization, and higher effluent concentrations than in monocomponent operation, indicating competitive adsorption for shared sites.

Scale-up substantially enhances the column performance by increasing the bed dimensions and hydraulic residence time, which improves pollutant–adsorbent interactions. As a result, characteristic and breakthrough times, AC, and UC increase markedly, while DOU approaches completion, reflecting improved bed efficiency. LUB remains comparable across scales, indicating the preservation of mass-transfer behavior and breakthrough dynamics. Additionally, scaled-up columns show reduced effluent mass and concentration and significantly higher treated volume. Although competitive effects persist in multicomponent systems, ZPJC maintains stable and effective removal performance, demonstrating robustness and practical applicability of scale-up design under realistic mixed-contaminant conditions.

### 3.8 Assessment of ZPJC Performance with Other Adsorbents from the Literature

Previous studies have explored the removal of TET and ACT primarily through batch systems,^11,39–48^ while relatively few investigations explored continuous-mode removal via columns.^9,13,49–54^ ZPJC demonstrated strong adsorption of TET and ACT (Table S9), outperforming many reported adsorbents in capacity, kinetics, and pH adaptability. Notable feature is its rapid adsorption rate, enabling faster equilibrium and efficient removal, which enhances ZPJC’s practical application in wastewater treatment. Table S10 further highlights its robustness in column operations. Importantly, ZPJC can simultaneously remove multiple pollutants, an ability rarely reported, making it a highly efficient and sustainable material for large-scale wastewater treatment mitigating emerging contaminants.

## 4 Conclusions

Competitive adsorption of ACT and TET was investigated in this study by using green-synthesized ZnO-biochar derived from *Prosopis juliflora* (ZPJC). ZPJC was characterized using various techniques, and its adsorption performance was assessed in both batch and fixed-bed column experiments for monocomponent (ACT or TET) and multicomponent (ACT + TET) systems. In batch experiments, under optimized conditions (60 min time, 6.5 pH, and 3 g/L ZPJC dose), maximum adsorption capacity was 5.27 mg/g for TET and 9.26 mg/g for ACT. Adsorption followed the pseudo-second-order kinetic model and was best described by the Langmuir isotherm, indicating chemisorption as the dominant mechanism. Adsorption of ACT and TET onto ZPJC occurs through hydrogen bonding, electrostatic interactions, and π–π stacking, and equilibrium analysis confirms monolayer chemisorption, supported by kinetic modeling, with SEM revealing pore filling as the additional adsorption mechanism. For fixed-bed column studies, the adsorption capacity was enhanced with increasing bed depth and flow rate but declined at higher concentrations. The Thomas model provided the best fit to experimental data. In batch systems, low $\frac{Q_{\mathrm{Multi}}}{Q_{\mathrm{Mono}}}$ ratio for TET (0.40) suggests antagonism due to site competition and steric effects, while ACT (1.14) showed mild synergism. In column systems, both ACT (0.92) and TET (0.88) exhibited antagonistic behavior. Scale-up design of the column demonstrated that ZPJC could be effectively employed as a sustainable adsorbent for pharmaceutical contaminant removal while simultaneously addressing issues of *Prosopis juliflora* proliferation, making it a viable solution for environmental remediation.

## Supplementary Material



## Data Availability

The data that support the findings of this study are provided in the manuscript and in the Supporting Information for Publication. All relevant data are publicly accessible with the published article.

## Notes

Adsorption performance in mono- and multicomponent systems; regeneration and reusability studies; proposed adsorption mechanisms; schematic illustrations; physicochemical properties of ACT and TET; kinetics, isotherm, and breakthrough model equations; column performance parameters and data analysis; error analysis of kinetic and isotherm models; BDST and competitive Langmuir constants; and comparative evaluation of adsorbents in batch and column systems (PDF)
