# Supplementary material for: Acetaminophen and Tetracycline Removal Using Prosopis juliflora-Derived ZnO-Modified Biochar: Evaluation in Batch and Continuous Systems
Source: ACS Omega. 2026 Mar 16;11(12):19180–91. doi: 10.1021/acsomega.5c12318 (PMC13044829; doi:10.1021/acsomega.5c12318)
Supplement: Supplementary file 1 [file ao5c12318_si_001.pdf]

Acetaminophen and Tetracycline Removal Using *Prosopis juliflora* - Derived ZnO-Modified Biochar: Evaluation in Batch and Continuous Systems

Manjunath Singanodi Vallabha[*a], Syeda Rabia Asma[a], Rajkumar Reddy[a], Bhojaraja Mohan[b], Chikmagalur Raju Girish[*b]

[a] *Department of Civil Engineering, BMS College of Engineering, Bangalore 560019, Karnataka, India*

[b] *Department of Chemical Engineering, Manipal Institute of Technology, Manipal Academy of Higher Education, Manipal 576104, Karnataka, India*

*Corresponding authors Email: manjunathsv.civ@bmsce.ac.in, cr.girish@manipal.edu

SUPPORTING INFORMATION FOR PUBLICATION

LIST OF FIGURES

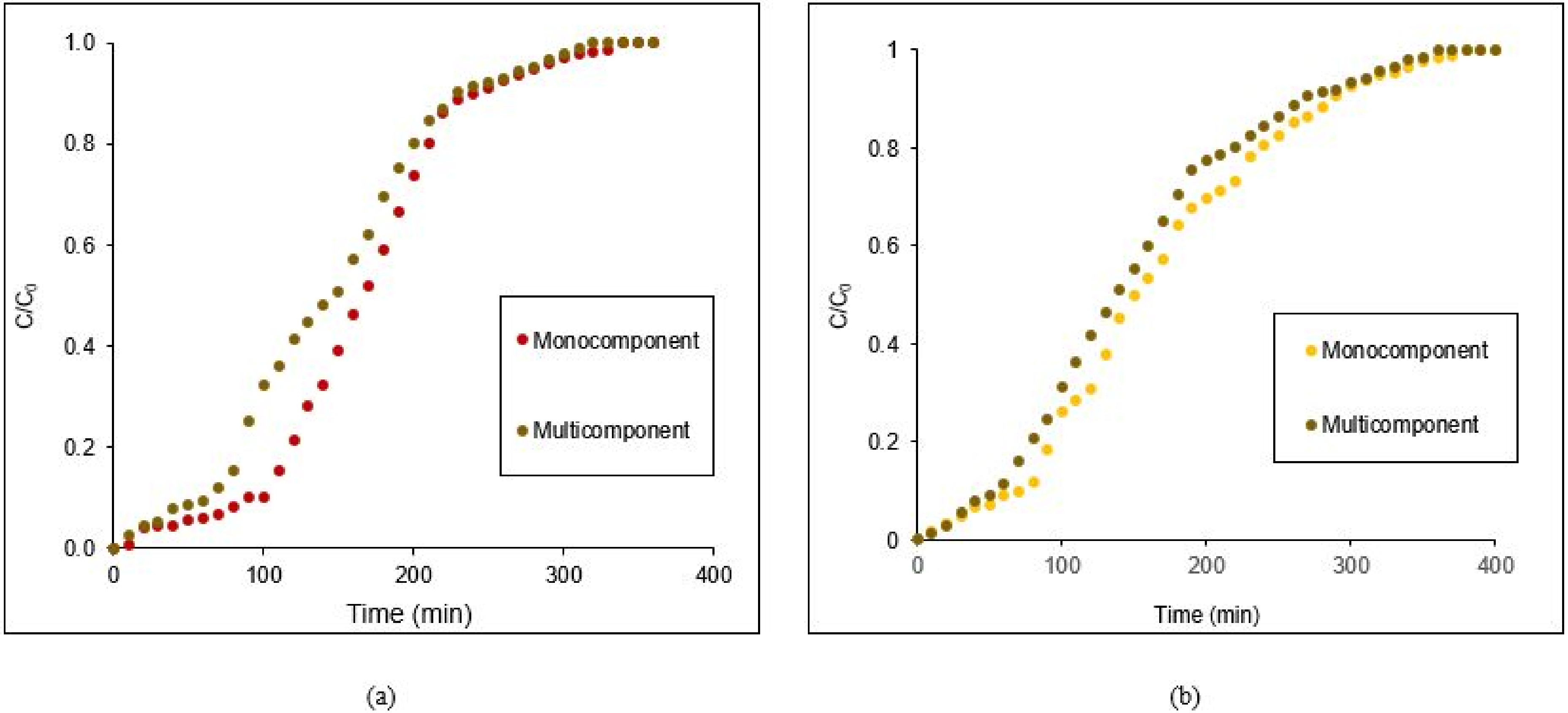

Fig. S1. Removal of TET and ACT from TET+ACT multi-component system using ZPJC: (a) TET and (b) ACT

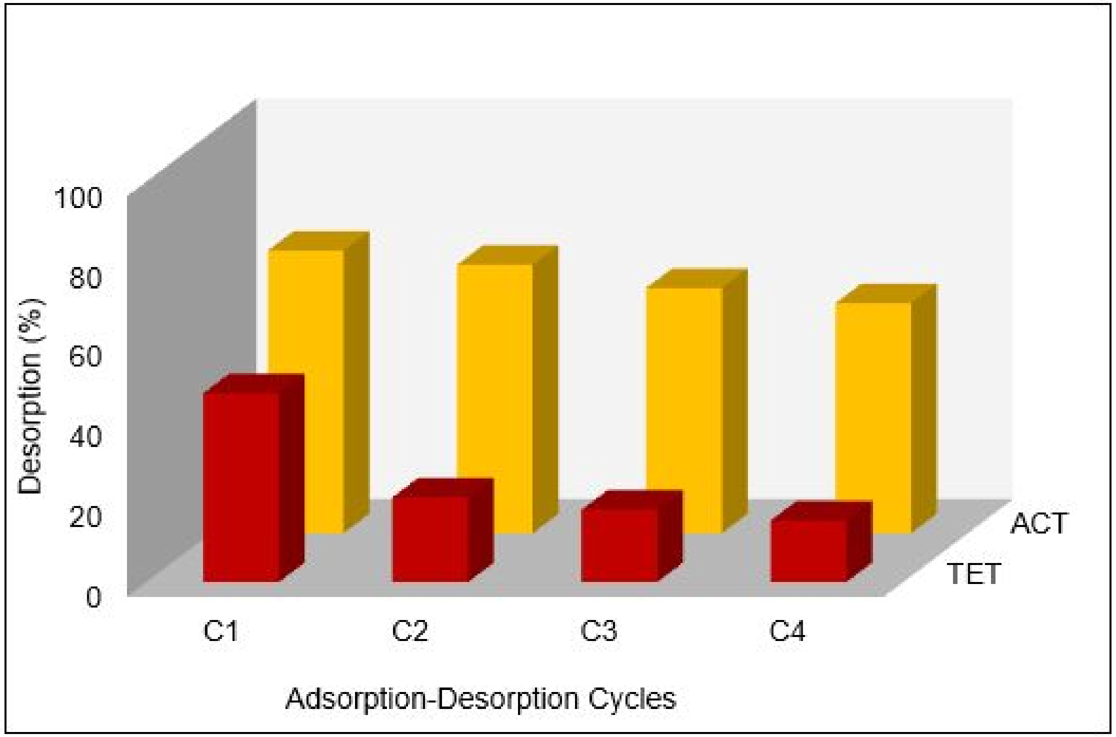

Fig. S2. ZPJC regeneration and reusability potential for TET and ACT

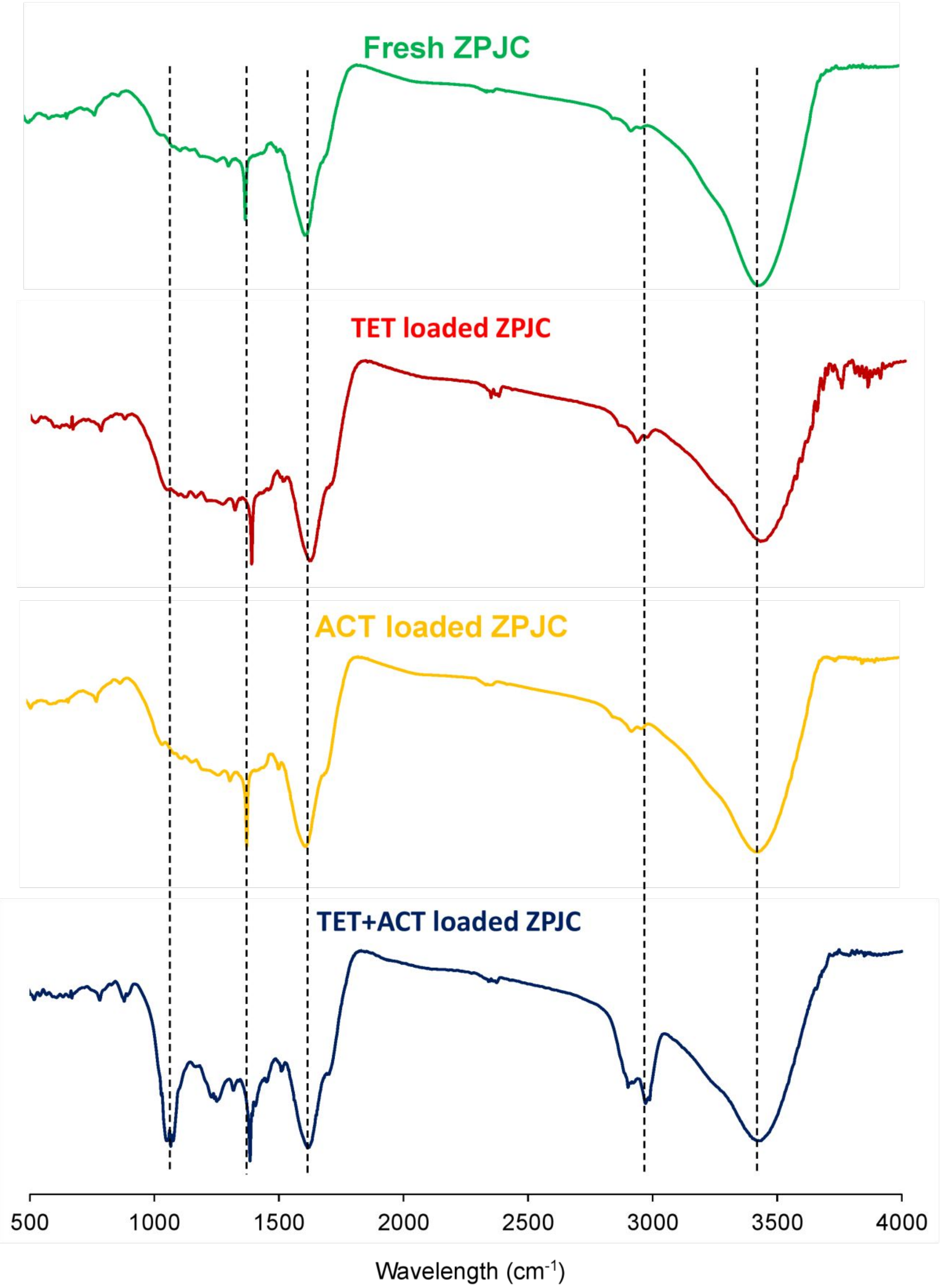

Fig. S3. Adsorption mechanism for removal of TET and ACT using ZPJC

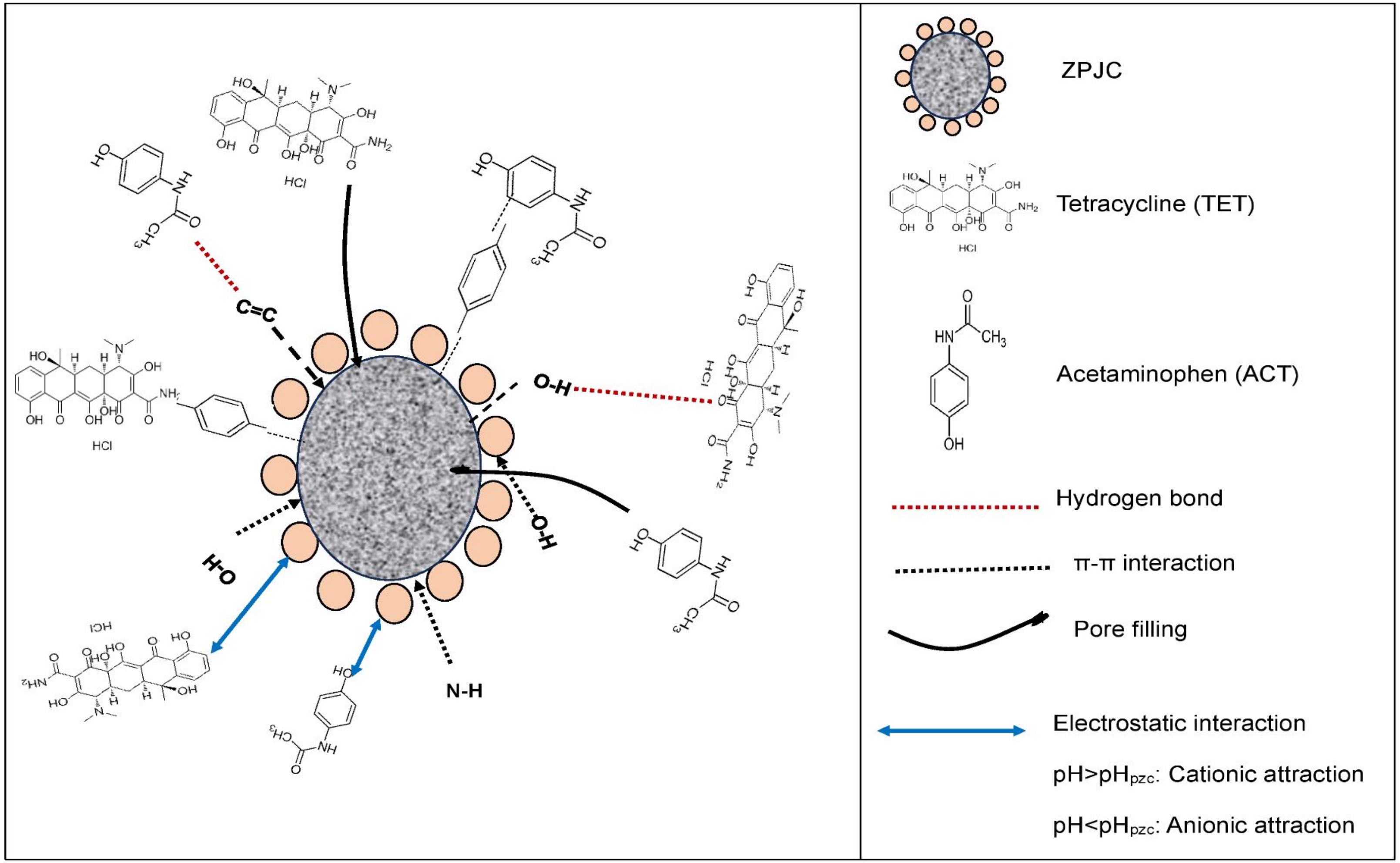

Fig. S4. Illustration of adsorption mechanism for removal of TET and ACT onto ZPJC

LIST OF TABLES

Table S1. Physicochemical and environmental properties of ACT and TET

Property	ACT	TET
Molecular formula	$C_8H_9NO_2$	$C_{22}H_{24}N_2O_8$
Molecular Weight (g/mol)	151.16	444.43
Chemical class	Analgesic and antipyretic	Broad-spectrum antibiotic
Chemical structure	Single aromatic ring with hydroxyl and amide groups	Polycyclic structure with multiple hydroxyl, amide and keto groups
pKa values	~9.5	~3.3,7.7 and 9.7
Solubility in water	High (~14 g/L at 25°C)	Moderate to high (231 mg/L at 25°C)
Polarity	Moderately polar	Highly polar and amphoteric
Persistence in environment	Moderately persistent	Highly persistent
Relevance to adsorption studies	Small molecular size, tests adsorbent affinity for low-molecular weight pollutants	Large, multifunctional molecule, tests surface interaction and complex adsorption mechanisms

Table S2. Kinetic and isotherm models

	Models	Expressions
Kinetic model	Pseudo-first-order	$ln(q_e - q_t) = ln\, q_e - K_1 t$
	Pseudo-second-order	$\frac{t}{q_t} = \frac{1}{K_2 q_e^2} + \frac{t}{q_e}$
	Intra-particle diffusion	$q_t = K_{ID} t^{1/2} + C$
	Liquid-film diffusion	$ln\left[1 - \frac{q_e}{q_t}\right] = -K_{FD} t + A$
	Elovich	$q_t = \frac{1}{\beta} ln\alpha\beta + \frac{1}{\beta} ln\, t$
2-parameter Isotherm model	Langmuir	$\frac{1}{q_e} = \frac{1}{q_m} + \frac{1}{q_m K_L C_e}$
	Freundlich	$ln\, q_e = lnK_F + {}^{1}/_{n}\, lnC_e$
	Temkin	$q_e = B\, ln\, A_T + B\, ln\, C_e$
	Dubinin-Radushkevich	$lnq_e = ln(q_m) - (K_{DR}\varepsilon^2)$
	Elovich	$ln\frac{q_e}{C_e} = lnK_E q_m - \frac{1}{q_m} q_e$
3-parameter Isotherm model	Redlich-peterson	$qe = \frac{K_{RP} C_e}{1 + \alpha_{RP} C_e^g}$
	Khan	$q_e = \frac{q_s b_K C_e}{(1 + b_K C_e)^{n_K}}$
	Sips	$q_e = \frac{q_{mS} K_S C_e^{m_S}}{1 + K_S C_e^{m_S}}$
	Hill	$q_e = \frac{q_{S_H} C_e^{n_H}}{K_D + C_e^{n_H}}$
	Toth	$q_e = \frac{K_T C_e}{\left[A_T + C_e^{T_T}\right]^{1/T_T}}$

Where, q_e and q_t are adsorption ability at equilibrium time (mg/g, mg/g respectively); K_1 and K_2 are pseudo-first-order rate constants (min^{-1} and g/mg/min, respectively); K_{ID} and C are intercept; K_{FD} and A are pseudo-second-order rate constants for liquid-film diffusion and liquid-film constant, respectively; α and β are constants of Elovich kinetic model.
Isotherm: C_e (mg/L) is isotherm concentration, q_e (mg/g) is adsorption capacity at equilibrium, q_m (mg/g) is maximum adsorption capacity, K_L (L/mg) is Langmuir constant, and n is Freundlich constant. A_T is Temkin equilibrium constant (L/mg), B is Temkin constant, K_{DR} (mol^2/kJ^2) is mean free energy of adsorption and $\varepsilon = RTln[1 + \frac{1}{C_e}]$ is polyani potential; K_E (L/mg) is Elovich equilibrium constant. K_{RP} is Redlich-Peterson isotherm constant (L/g); b_K is Khan isotherm constant; q_{mS} is Sips adsorption capacity (mg/g); q_{S_H} is Hill isotherm maximum uptake saturation (mg/L); K_T is Toth isotherm constant.

Table S3. Breakthrough models for prediction of column data

Breakthrough models	Expression
Thomas model	$ln\left(\frac{C_o}{C}-1\right)=\frac{k_{TH}\times Q_{TH}\times W}{Q}-k_{TH}\times C_o\times t$
Adams-Bohart model	$ln\left(\frac{C}{C_o}\right) = k_{AB}\times C_o\times t-k_{AB}\times N_{AB}\left(\frac{Z}{u}\right)$
Yoon-Nelson model	$ln\left(\frac{C}{C_o-C}\right)=k_{YN}t-k_{YN}\tau_{0.5}$
BDST model	$t=\left(\frac{N_o}{C_o\times U_o}\right)Z-\left(\frac{1}{k\times C_o}\right)ln\left(\frac{C_o}{C}-1\right)$

Note: Q_{TH} is adsorption capacity (mg/g), k_{TH} is Thomas rate constant (mL/mg min), C is concentration of adsorbate at time t (mg/L), C_o is initial adsorbate concentration (mg/L), W is total dry weight of adsorbate in column (mg), Q is volumetric flow rate, N_{AB} is saturation concentration (mg/L), k_{AB} is Adams-Bohart kinetic constant (L/mg.min), u is linear flow velocity (cm/min), Z is depth of column bed (m), $\tau_{0.5}$ is time necessary for 50% adsorbate breakthrough (min), k_{YN} is Yoon-Nelson kinetic rate constant (L/min), t_b is service time at breakthrough point (min), N_o is adsorption capacity of bed (mg/L) and k_B is rate constant (L/mg min).

Table S4. Adsorption parameters from column studies

Parameter	Expression
Mass retained (m_{ads}, mg)	$m_{ads} = \frac{QA}{1000} = \frac{Q}{1000}\int_{t=0}^{t=t_{total}} C_{ads}dt$
Equilibrium sorption capacity (q_e in mg/g)	$q_e = \frac{q_{total}}{W}$
Total adsorbate mass entering column (m_{total}in mg)	$m_{total} = \frac{C_o Q t_{total}}{1000}$
Total effluent volume (V_{eff} in mL)	$V_{eff} = Q\ t_{total}$
Empty bed contact time (EBCT in min)	$EBCT = \frac{V_B}{Q}$
Removal efficiency (R in %)	R (%) $= \frac{m_{ads}}{m_{total}}$ x 100
Mass transfer zone (MTZ in cm)	$MTZ = H\left(\frac{t_e - t_b}{t_e}\right)$

Note: A (cm^2) *represents area under breakthrough curve,* t_{total} (min) *denotes total flow time and Q (mL/min) is flow rate. Adsorbed concentration* C_{ads} *(mg/L) was obtained as* $C_o - C_t$*. W represents total dry mass of ZPJC used in column,* V_B *is volume of adsorbent bed and H is bed height. Breakthrough time* (t_b, min) *corresponds to point where effluent concentration reaches 10% of initial concentration, expressed as* $\frac{C}{C_0} = 0.1$*. Exhaustion time* (t_e, min) *is defined as point where* $\frac{C}{C_0} = 0.9$.

Table S5. Error analysis for kinetic and isotherm parameters

Type	Models	Constants	TET	ACT
Kinetic models	Pseudo-first-order	$RMSE$	0.4246	0.3558
		X^2	0.4794	0.3372
		R^2	1.0000	0.9850
	Pseudo-second-order	$RMSE$	0.0618	0.1060
		X^2	0.0096	0.0281
		R^2	1.000	0.999
	Intra-particle-diffusion	$RMSE$	0.0225	0.0667
		X^2	0.0013	0.0107
		R^2	0.9960	0.9540
	Liquid-film-diffusion	$RMSE$	0.8973	0.8694
		X^2	2.1433	2.0288
		R^2	0.992	0.995
	Elovich	$RMSE$	0.0292	0.4109
		X^2	0.0022	0.4459
		R^2	0.993	0.924
Two-parameter isotherm models	Langmuir	$RMSE$	0.0782	0.1224
		X^2	0.1048	0.2416
		R^2	0.998	1.000
	Freundlich	$RMSE$	0.5164	0.3183
		X^2	0.9228	0.4723
		R^2	0.990	0.992
	Temkin	$RMSE$	1.0522	0.5276
		X^2	185.0475	12.1680
		R^2	0.996	0.999
	Dubinin-Radushkevich	$RMSE$	0.0747	0.1010
		X^2	0.1608	0.2398
		R^2	0.981	0.981
	Elovich	$RMSE$	0.0698	0.0996
		X^2	0.1327	0.2454
		R^2	0.970	0.970
Three-parameter isotherm models	Redlich-Peterson	$RMSE$	0.0271	0.0960
		X^2	0.0145	0.2348
		R^2	0.999	0.961
	Khan	$RMSE$	0.0736	0.1545
		X^2	0.1439	0.3682
		R^2	0.962	0.979
	Sips	$RMSE$	0.0267	0.0482
		X^2	0.0104	0.0497
		R^2	0.999	0.989
	Hill	$RMSE$	0.0598	0.0906
		X^2	0.1128	0.2211
		R^2	0.976	0.969
	Toth	$RMSE$	0.0598	0.0906
		X^2	0.1128	0.2211
		R^2	0.980	0.971

Table S6. Column data analysis for adsorptive removal of TET and ACT employing ZPJC

System	Adsorbate	H (cm)	Q (L/h)	C_0 (mg/L)	m (g)	t_b (min)	t_t (min)	V_b (mL)	V_t (mL)	m_{total} (mg)	m_{ads} (mg)	q_{e-exp} (mg/g)	R (%)	EBCT (min)	MTZ (cm)
Mono-component (TET/ACT)	TET	3	1	2.5	0.37	30	210	500	2167	5.42	3.11	8.70	57.38	0.141	2.57
		6	1	2.5	0.71	90	340	1500	4167	10.42	6.89	9.64	66.11	0.283	4.26
		9	1	2.5	1.07	160	450	2667	5833	14.58	10.34	9.66	70.92	0.424	5.65
		6	0.5	2.5	0.71	130	520	1083	2750	6.88	4.88	6.83	70.94	0.566	3.55
		6	2	2.5	0.71	30	260	1000	4333	10.83	6.93	9.70	63.94	0.141	4.80
		6	1	1	0.71	60	430	1000	5000	5.00	3.42	4.79	68.41	0.283	3.96
		6	1	5	0.71	30	300	500	2833	14.17	8.29	11.61	58.54	0.283	4.91
	ACT	3	1	2.5	0.37	20	240	333	3000	7.50	4.10	11.49	54.69	0.141	2.67
		6	1	2.5	0.71	80	380	1333	4833	12.08	6.84	9.59	56.65	0.283	4.34
		9	1	2.5	1.07	160	480	2667	6500	16.25	10.71	9.99	65.88	0.424	5.31
		6	0.5	2.5	0.71	150	540	1250	2000	5.00	4.05	5.66	80.91	0.566	2.25
		6	2	2.5	0.71	30	260	1000	6333	15.83	8.63	12.08	54.48	0.141	5.05
		6	1	1	0.71	170	550	2833	7500	7.50	4.45	6.23	59.36	0.283	3.73
		6	1	5	0.71	30	270	500	2833	14.17	7.86	11.00	55.45	0.283	4.94
Multi-component (TET+ACT)	TET	6	1	2.5+2.5	0.71	70	320	1167	3833	9.58	6.01	8.41	62.68	0.283	4.17
	ACT	6	1	2.5+2.5	0.71	60	360	1000	4500	11.25	6.20	8.69	55.15	0.283	4.67

Table S7. BDST model constants

Breakthrough time (t_b)	Bed depth (cm)	TET		ACT	
		$t_b - exp$ (min)	$t_b - cal$ (min)	$t_b - exp$ (min)	$t_b - cal$ (min)
10%	3	30	16	20	11
	6	90	49	80	33
	9	160	114	160	97
	Constants	N_o:1149, K:0.010, R^2 :0.998		N_o :1238, K: 0.012, R^2 :0.993	

Table S8. Langmuir competitive model constants for multi-component adsorption system

Multi-component system	Pollutant	Q_{Multi} (mg/g)	K_L	$\frac{Q_{Multi}}{Q_{Mono}}$	R^2
TET+ACT	TET	2.13	0.823	0.40	0.9905
	ACT	10.53	0.579	1.14	0.9989

Note: $\frac{Q_{Multi}}{Q_{Mono}} < 1$ indicates antagonistic effect; $\frac{Q_{Multi}}{Q_{Mono}} > 1$ indicates synergistic effect; $\frac{Q_{Multi}}{Q_{Mono}} = 1$ indicates no interaction

Table S9. Evaluation of various adsorbents in removing TET and ACT in batch system

Adsorbate	Adsorbent	Experimental conditions				Kinetic model	Isotherm model	Adsorption capacity (mg/g)	References
		T (min)	Dose (g/L)	C_0 (mg/L)	pH				
TET	Mineral pumice	1500	4	1-60	3-9	Pseudo-second order	Langmuir	3.35	(Lu et al., 2018)
	Poplar Biochar (RBC 300)	264 h	1	10-50	3-9	Elovich	Freundlich	4.30	(Huang et al., 2017)
	Bovine manure derived biochar	720	4	10	3-11	Pseudo-second order	Langmuir	5.38	(Zhao et al., 2021)
	Iron coated carbonized humic acid	640	2	5-400	2-10	Pseudo-second order	Langmuir	12.27	(Xie et al., 2020a)
	$CuCoFe_2O_4$@chitosan	20	0.4	5	3.5	Pseudo-second order	Freundlich	1.52	(Nasiri et al., 2022)
	Activated granular sludge	800	2	5-150	3-9	Pseudo-second order	Langmuir	2.984	(Li et al., 2013)
	ZPJC (mono-component)	60	3	0.1-10	3-11	Pseudo-second order	Langmuir	5.27	This study
	ZPJC (multi-component)	60	3	0.1-10	3-11	Pseudo-second order	Langmuir	2.13	This study
ACT	Azadirachta indica induced ZnO	30	2	30	2-12	Pseudo-second order	Langmuir	7.87	(Sanjeev et al., 2023a)
	Molecularly imprinting polymer	20	0.1	0-10	1-6	-	Langmuir	0.35	(Lee et al., 2017)
	Oil palm fibre biochar	60	1.0	0.4-40	4-12	Pseudo-first-order	Langmuir	7.30	(Hethnawi et al., 2020)
	Gundelia tournefortee activated carbon	30	0.25	70-120	3-9	Pseudo-second-order	Freundlich	4.25	(Mokhtaryan et al., 2023a)
	Amine hexagonal mesoporous silica	600	2	0.04-0.3	5-9	Pseudo-second order	Langmuir	0.15	(Suriyanon et al., 2015)
	ZPJC (mono-component)	60	3	0.1-10	3-11	Pseudo-second order	Langmuir	9.26	This study
	ZPJC (multi-component)	60	3	0.1-10	3-11	Pseudo-second order	Langmuir	10.53	This study

Table S10. Evaluation of various adsorbents in removing TET and ACT using adsorption column system

Adsorbate	Adsorbent	Operational parameters H (cm)	C_o (mg/L)	Q (mL/min)	Breakthrough model	Adsorption capacity (mg/g)	References
TET	Chitosan coated coconut shell activated carbon	6.5-14.5	49.2-51.8	1-2	Thomas	4.75	(Yaqubi et al., 2021a)
	Moso bamboo charcoal	2-6	20-80	3.3-10	Adams-Bohart	19.4	(Liao et al., 2013)
	Granular activated carbon	1	80	6	Yan	2.08	(de Oliveira et al., 2023)
	Mesoporous activated carbon	2-6	20-80	4-8	Yoon-Nelson	7.19	(Marzbali and Esmaieli, 2017)
	Commercial grade activated carbon	4.5-10	0.2-0.6	1.5-5	Thomas and Yoon-Nelson	0.069	(Swapna Priya and Radha, 2016)
	ZPJC (mono-component)	3-9	1-5	8.33-33.33	Thomas	9.64	This study
	ZPJC (multi-component)	3-9	1-5	8.33-33.33	Thomas	8.50	This study
ACT	KOH *Parthenium hysterophoru*s biochar (KPC)	3-9	5-20	0.25-1	Thomas	10.42	(Manjunath et al., 2024)
	Sugarcane bagasse carbon	20-35	40-80	1.5-3	-	0.24	(Juela, 2020)
	Chitosan coated granular activated carbon	25-45	40-120	2-6	Thomas and Yoon-Nelson	16.67	(Yanyan et al., 2018)
	Corn cob carbon	20-35	40-80	1.5-3	-	0.28	(Juela, 2020)
	ZPJC (mono-component)	3-9	1-5	8.33-33.33	Thomas	9.59	This study
	ZPJC (multi-component)	3-9	1-5	8.33-33.33	Thomas	9.05	This study
